# Modulation of the immune response and infection pattern to *Leishmania donovani* in visceral leishmaniasis due to arsenic exposure: An *in vitro* study

**DOI:** 10.1371/journal.pone.0210737

**Published:** 2019-02-05

**Authors:** Ghufran Ahmed, Ajit K. Thakur, Pushpanjali, Snehil, Sanjay K. Chaturvedi, Pushkar Shivam, Fauzia Jamal, Manish K. Singh, Sanjiva Bimal, Subhankar K. Singh, Sunil K. Choudhary, Pradeep Das, Shyam Narayan

**Affiliations:** 1 Division of Microbiology, Rajendra Memorial Research Institute of Medical Sciences (Indian Council of Medical Research), Agamkuan, Patna, Bihar, India; 2 Division of Immunology, Rajendra Memorial Research Institute of Medical Sciences (Indian Council of Medical Research), Agamkuan, Patna, Bihar, India; 3 University Department of Botany, Tilka Manjhi Bhagalpur University, Bhagalpur, Bihar, India; 4 Division of Molecular Biology, Rajendra Memorial Research Institute of Medical Sciences (Indian Council of Medical Research), Agamkuan, Patna, Bihar, India; Taibah University, SAUDI ARABIA

## Abstract

The arsenic contamination of ground water in visceral leishmaniasis (VL) endemic areas in Bihar, India leads to human exposure through drinking water. Possibly, the consumed arsenic (As) accumulates in the tissues of VL patients, who subsequently internalize intracellular amastigotes to confer resistance against chemotherapy to the parasite, leading to modulation in the host’s immune response. This hypothesis appears to be consistent with the *in vitro* findings that in arsenic-exposed parasites, the mitochondrial membrane potential became depolarized, whereas the reduced thiol and lactate production was overexpressed with enhanced glucose consumption; therefore, the reduced thiol possibly supports an immunosuppressive state in the host cells. This observation was well supported by the down-regulated expression of pro-inflammatory cytokines (IL-2, IL-12, IFN-γ, and TNF-α) with a suppressed anti-leishmanial function of macrophage (NO, ROS). In contrast, the pathophysiological mechanism of VL has received ample support by the promotion of Th2 cytokines (IL-4 and IL-10) in the presence of arsenic-exposed *Leishmania* parasites (Ld^AS^). Dysfunction of mitochondria and the overexpression of lactate production raise the possibility of the Warburg effect being operative through the up-regulation of glucose consumption by parasites to enhance the energy production, possibly augmenting virulence. Therefore, we surmise from our data that arsenic exposure to *Leishmania donovani* modulates the immune response and infection pattern by impairing parasite function, which may affect the anti-leishmanial effect in VL.

## Introduction

The *Leishmania* species are obligate intracellular protozoa of the kinetoplastids family that cause various clinical manifestations of leishmaniasis. The most severe form is visceral leishmaniasis (VL) caused by *Leishmania donovani*, which is fatal if left untreated [1]. Although leishmaniasis is widespread in 98 countries and more prevalent in tropical and sub-tropical regions, ≥90% of VL cases are reported from six countries, Bangladesh, Brazil, Ethiopia, India, Sudan and South Sudan, with a disease burden of 0.2–0.4 million and an annual mortality rate of 0.02–0.04 million worldwide [2,3,4]. The emergence of drug resistance to most of the available anti-leishmanial drugs has created a new level of obstacles in the treatment of VL [5]. The widespread distribution of arsenic in the ground water of VL endemic areas is one of the major contributing factors to antimony resistance [6]. Arsenic is a highly toxic metalloid, released into the water of Indian subcontinent, and its co-existence with antimony resistance in the VL endemic area of Bihar, India has led to multiple challenges for public health [7,8]. The consumption of arsenic contaminated water by the inhabitants of this region could have resulted in the induction of changes in parasites through arsenic exposure in their hosts. Arsenic is toxic to most cells, including microorganisms that have evolved multiple mechanisms to detoxify the effect of arsenic and have survived well in such harsh environmental conditions [9,10]. In connection to arsenic entry in parasites, arsenic exposure induces resistance in *Leishmania* parasites [6] as arsenicals and antimonials are transported by the same aquaglyceroporin (AQP1) channels [11,12] of microbes. It has previously been reported that the level of thiol has been increased several fold in arsenic-exposed and antimony-resistant *Leishmania* parasites that bind to and are sequestered in intracellular vesicles [13,14]. The mitochondria have a pivotal role in adenosine triphosphate (ATP) generation, and arsenic exerts its toxic effect directly on mitochondria. The metalloid lowers the rate of the Krebs cycle and alters the expression of most of the mitochondrial enzymes to depolarize the membrane potential and cause oxidative phosphorylation [15,16].

In the case of *Caenorhabditis elegans*, arsenic exposure induces severe mitochondrial dysfunction and alters pyruvate metabolism. Low-dose arsenic exposure induces the Warburg effect by shifting from mitochondrial oxidative phosphorylation to aerobic glycolysis [17,18]. As the effect of arsenic exposure on the mitochondria of *Leishmania* is poorly understood, this particular parameter was examined. The effect of arsenite exposure on cancer cell lines has clearly shown that the intracellular concentration of lactate production is up-regulated in a dose-dependent fashion, leading towards the Warburg effect to generate more energy for their survival [17,18,19]. Arsenic exposure also exerts immunotoxicity by diminishing T cell proliferation and modulating the level of secreted cytokines (TNF-α, IFN-γ, IL-2, IL-4, IL-5, IL-6, IL-10, IL-12) [20,21]. Arsenic exposure also inhibits the activation of the defence of macrophages by diminishing the production of reactive oxygen species (ROS) and nitric oxide (NO) to weaken the host defence mechanism and render the host more susceptible to invading pathogens [22,23]. Although the effect of arsenic on the immune system and infection is specific, the impact of arsenic-induced *Leishmania* parasites in the modulation of the immune system of healthy host cells and infection pattern is not yet known. Therefore, the change in *Leishmania* biology after arsenic exposure and its occurrence in the alteration of the immune system and infection of healthy cells are reported in an *in vitro* study.

## Materials and methods

### Institutional animal ethics approval

The Institutional Animal Ethics (IAE) committee of ICMR-Rajendra Memorial Research Institute of Medicals Sciences (letter No. RMRI/ICMR/AH/416/2017-18) had approved this study prior to start the work on experimental animals and their tissues and cells.

### Parasite culture and serial arsenic exposure to parasites

The reference strain AG83 (MHOM/IN/1983/AG83) of *Leishmania donovani* (Ld), procured from the cryobank of the RMRIMS (ICMR) repository was maintained routinely in RPMI-1640 medium (Sigma-Aldrich), supplemented with 10% heat-inactivated foetal bovine serum (FBS) with 50 U penicillin (Sigma-Aldrich), 50 μg streptomycin, and 25 μg gentamicin/ml by incubating to facilitate biological oxygen demand (BOD) at 25°C. The metacyclic promastigotes were harvested by Ficoll gradient [24]. A portion of the procured promastigotes was exposed to sodium (meta) arsenite (lot#SLBJ2560V) (Sigma-Aldrich) dissolved in Milli Q water at a concentration between 0.5 and 1.5 mg/L and maintained *in vitro* by passaging every 3–4 days.

### Assessment of infection pattern

For the assessment of the pathogenicity and infectivity of Ld^AS^ and Ld, mouse peritoneal macrophages were isolated as previously described [25] with minor modification. Peritoneal exudate cells (PECs) were aspirated in a sterile manner from BALB/c mouse peritoneum after treatment with 2% starch solution for 24 hrs and centrifuged at 500 g for 10 minutes at 4°C. The cells were washed twice with phosphate buffered saline (PBS) and were suspended in complete RPMI-1640 medium. The cell number was adjusted (0.5 to 0.6 x 10^6^ cells/ml) using a Neubauer counting chamber (Fein Optic, Jena, Germany). The cells were cultured in a Lab-Tek^R^ chamber slide (Nunc^TM^) and incubated in a 5% CO_2_ incubator with humidified atmosphere (95%) at 37°C for 48 hrs. The cultured peritoneal macrophage cells (MΦ) were challenged with the metacyclic form of Ld^AS^ and Ld promastigotes at a ratio of 1:10 and further incubated in a CO_2_ incubator. The non-internalized parasites were removed by gentle washing with RPMI-1640 medium after 4 hrs of incubation, and the culture plates were further incubated for the next 48 hrs [24]. The intracellular parasitic load was determined microscopically in Ld^AS^- and Ld-challenged wells.

### Assessment of sodium antimony gluconate (SAG) effect

The effect of sodium antimony gluconate (SAG) on Ld^AS^ and Ld viability was monitored by microscopy and MTT assay (3-(4,5-dimethylthiazol-2-yl)-2,5-diphenyltetrazolium bromide) (Sigma-Aldrich) as previously described [26] with minor modification. For this, metacyclic promastigotes were harvested and washed with PBS. The culture was re-suspended in complete RPMI-1640 medium (Sigma-Aldrich), and the parasite number was adjusted (1x10^6^/ml) to infect the PECs and seeded in the Lab Teck^R^ chamber (Nunc). The different concentrations (20, 40, 60, 80, 100, 120, 140 and 150 μg/ml) of SAG (Albert David, India) were added to the appropriate wells of arsenic-exposed and unexposed intracellular parasite-infected macrophages. The untreated well of parasites served as the control subject. The culture Lab Teck^R^ chamber was incubated in a humidified CO_2_ incubator at 37°C for 72 hrs. After incubation, Lab Teck^R^ chamber was fixed in absolute methanol and stained with giemsa for microscopic analysis. For the MTT assay, 10% MTT reagent was added into the culture medium and incubated for 4 hrs. An equal amount of MTT solubilizing agent was added. The final culture was transferred to a 96 well plate and mixed well, and the optical density (OD) was measured on a spectrophotometer (BIO-RAD) at 570 nm.

### Evaluation of intracellular reduced thiol

The level of total intracellular reduced thiol was measured in deproteinized extracts of metacyclic Ld^AS^ and Ld promastigotes as previously described [27] with minor modification. Briefly, the *L*. *donovani* culture was harvested, washed twice in buffer (0.14 M Na_3_PO_4_, 0.14 M K_3_PO_4_, 0.14 M NaCl and 3 mM KCl), suspended in 0.6 ml of 25% trichloroacetic acid and freeze thawed for one cycle. The denatured proteins and cell debris were removed by centrifugation. The thiol content in the supernatant was measured with 0.6 mM 5,5-dithio-bis 2-nitrobenzoic acid (DTNB, Ellman’s reagent) in 0.2 M Na_3_PO_4_ buffer (pH 8.0). The concentration of the DTNB derivatives of thiol was measured spectrophotometrically at 412 nm.

### Measurement of mitochondrial membrane potential (Δψm)

Alteration in the mitochondrial membrane potential of *L*. *donovani* was measured using (JC-1) dye (Invitrogen), as previously reported [28,29]. Briefly, Ld^AS^ and Ld metacyclic promastigotes (1x10^6^/ml) were harvested, washed and incubated with 10 μM JC-1 dye at 37°C for 10 minutes. After incubation, the parasites were washed and re-suspended in RPMI-1640 medium followed by fluorescence measurement by flow cytometry (FACS Calibur^TM^, Becton Dickinson, San Diego, USA) with excitation at 488 nm and emission at 530/585 nm for green and red channels, respectively. Data were analysed using FACS Calibur Cell Quest Pro^TM^ software for the mean green and red fluorescence. The ratio of red and green fluorescence (i.e., 585/530) determined the Δψm.

### Lactate assay

The intracellular lactate was measured using a lactate assay kit (lot# B1K190607V) according to the manufacturer’s instructions (Sigma-Aldrich) as previously described [18]. Briefly, the metacyclic promastigotes of Ld^AS^ and Ld at the density of 2x10^7^/ml were harvested and washed with PBS. After washing, the cultures were homogenized with lactate assay buffer followed by centrifugation at 13,000 g for 10 minutes to remove the lactate dehydrogenase using a 10 kDa molecular weight cut-off (MWCO) spin filter. The lactate present in the soluble fraction was estimated spectrophotometrically at 570 nm. The data were analysed by comparison with a standard curve generated as per the manufacturer's guidelines, and the results were represented in a bar diagram.

### Glucose assay

To measure the glucose consumption of Ld^AS^ and Ld parasites, the metacyclic promastigotes of *Leishmania* parasites (5x10^8^/ml) were harvested, washed with PBS and suspended in a fresh glucose-free M199 medium supplemented with 10 mM glucose for 24 hrs [30]. A quantity of 200 μl culture was taken from the glucose-free medium and centrifuged at 700 g for 10 minutes to pellet the parasites. The glucose concentration in the culture supernatant was estimated with a glucose assay kit as per the manufacturer's instruction (Crest Biosystems, Goa, India).

### Nitrite assay

The PECs of BALB/c mouse-derived macrophages (3x10^6^ cells/ml) were cultured at 37°C in an incubator with 5% CO_2_ and 95% humidity. The cells were stimulated with the metacyclic form of Ld^AS^ and Ld promastigotes at a ratio of 10:1 and incubated at 37°C in an incubator with 5% CO_2_ for 48 hrs. The cell supernatant was collected by centrifugation at 500 g for 10 minutes, and the NO was measured in the samples using Griess reagent [31]. The absorbance was recorded at 540 nm (BIO-RAD), and the amount of NO was estimated by comparison with a standard curve.

### Estimation of reactive oxygen species (ROS)

The ROS was estimated in mouse macrophages as previously described [32] with a few modifications. The cells were collected and triggered with metacyclic form of Ld^AS^ and Ld promastigotes experimentally and with lipopolysaccharide (LPS, 100 ng/ml) as a positive control. The cells without stimulation served as a negative control. All samples were incubated in a water bath at 37°C for 10 min followed by stimulation with 10 mM dihydrorhodamine (DHR) and re-incubation for 15 min to allow the internalization of DHR in the cells to react with the oxidative burst species produced by the stimulated cells. The cells were washed with PBS followed by centrifugation at 300 g for 10 min. Finally, the supernatant was decanted, and 300 μl PBS was added. The production of ROS in stimulated cell samples was measured as the mean fluorescence intensity (MFI) using flow-cytometer FACS Calibur equipped with Cell Quest Pro software (Becton Dickinson, San Diego, USA).

### Splenocytes for immunological response

The spleen cell homogenates were collected in Falcon tubes (Tarson) followed by centrifugation at 500 g for 10 minutes. A volume of 200 μl RBC lysis buffer (BD Biosciences) (1x) was added to the corresponding tubes and incubated at 37°C for 30 minutes. After incubation, the lysed cells were centrifuged and washed with phosphate buffer saline (PBS) to remove RBC lysates. Purified splenocytes were collected, cultured and stimulated with Ld^AS^ and Ld followed by incubation for 48 hrs in a CO_2_ incubator at 37°C with 5% CO_2_ for immunological purposes.

### Evaluation of intracellular cytokines

The intracellular cytokines were determined by flow cytometry of mouse splenocytes as previously described with minor modifications [33,34]. Briefly, the collected mouse splenocytes (1x10^6^/ml) from lymphoid organs were cultured and stimulated with Ld^AS^ and Ld metacyclic promastigotes incubated in a humidified CO_2_ incubator at 37°C. The culture was treated with GolgiStop (1 μg/ml) (BD Biosciences, USA) prior to harvesting 4 hrs later. After incubation, the cultured plate was kept on ice, and the adherent cells were scraped out from the culture plate and collected in a Falcon tube (BD Biosciences). The cells were washed with PBS and centrifuged at 300 g for 5 minutes, followed by staining with FITC-labelled anti-CD14^+^ antibodies (BD Biosciences). Subsequently, for CD4^+^ T cell analysis, non-adherent cells from another set of cell cultures were collected in Falcon tube and washed with PBS followed by staining with PerCP-labelled anti-CD4^+^ antibodies (BD Biosciences). The fluorescence-stained cells were incubated in Cytofix/Cytoperm solution for 30 minutes at 4°C and were washed with Perm Wash buffer (1x). The cells were re-stained with intracellular FITC-labelled anti-IL-2 antibodies, Alexa Fluor-labelled anti-IFN-γ antibodies, PE-labelled anti-IL-4 antibodies, PE-labelled anti-IL-10 antibodies, PE-labelled anti-IL-12 antibodies, or PE-labelled anti-TNF-α antibodies (BD Biosciences) and incubated for 30 minutes at 4°C. The cells were washed with staining buffer (PBS, 2% FCS, 0.09% NaN3, pH 7.2) and re-suspended in 300 μl staining buffer before being analysed on a flow-cytometer FACS Calibur (Becton Dickinson, San Diego, USA) equipped with Cell Quest Pro software.

### Determination of extracellular cytokines

The splenocytes of mice were collected, cultured [35] and stimulated with metacyclic Ld^AS^ and Ld promastigotes with the incubation at 37°C in a CO_2_ incubator. The supernatant was collected and centrifuged at 500 g for 10 minutes. The quantitative estimation of cytokines IL-10, IL-12 and IFN-γ was performed using an enzyme-linked immunosorbent assay kit (ELISA) (BD OptEIA^TM^ Elisa kit) as per the manufacturer’s protocol. The cytokine levels were detected by comparison with standard curves generated according to the manufacturer’s recommendations and are shown in a scatter diagram.

### Statistical analysis

The data are expressed as mean ± SEM (standard error of the mean), and statistical analysis was performed using Graph pad prism-5 (USA) software. The significance was determined by Student’s t-test and one-way analysis of variance (ANOVA) with *Tukey’s post hoc* multiple comparison tests. All experiments were run in triplicates and P value ≤ 0.05 considered significant.

## Results

### Arsenic exposure exacerbates the infection and resistance to SAG

**Fig 1:**
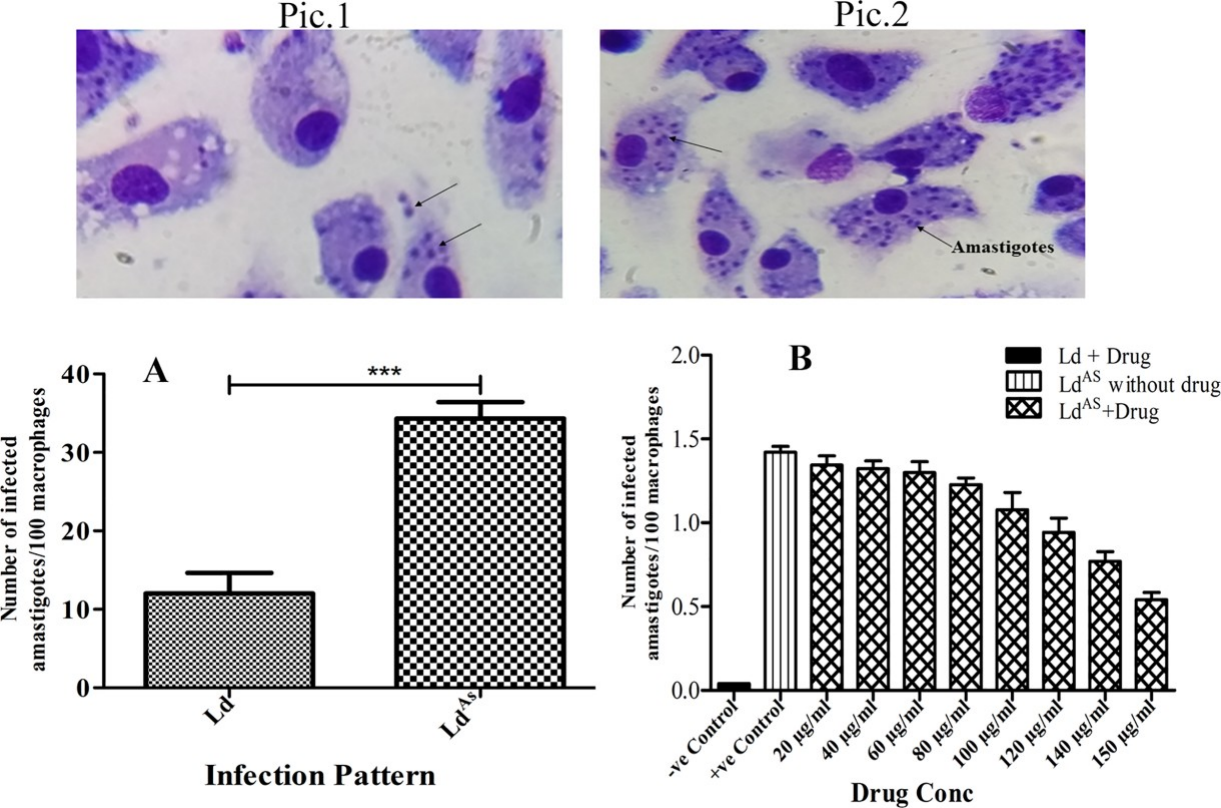
The macrophage cells, challenged with arsenic unexposed (Ld) and exposed (Ld^AS^) parasites of same strain, showed an infection pattern of 1.78x10^2^±50 and 5.43x10^2^±91 amastigotes/100 cells (100x), (Pic 1 and Pic 2) respectively. The percentage of their infectivity is plotted in bar diagram (***P≤0.001) (A), and their survivability against different concentration of SAG compared to negative (Ld with drug) and positive (Ld^AS^ without drug) controls (B) is also calculated. The data are expressed as mean ± SEM (n = 5) of independent experiment in triplicate and significance was determined by paired t test, one-way ANOVA with *Tukey’s post hoc* multiple comparison tests.

Most of the previous studies did not show the pathophysiological relevance of the arsenic exposure related changes in *Leishmania* parasites and their host cells, thus the pathophysiological consequences of arsenic related to VL and other infectious diseases are still remain poorly understood. The experiment was initiated to examine the impact of arsenic exposure on infectivity and viability of *Leishmania* parasite using microscopic and MTT assay techniques respectively. The Ld^AS^ was found to be more infective and virulent as the parasitic load was 3-fold higher (p <0.004) in macrophages challenged with Ld^AS^ than that with Ld (Fig 1A). Furthermore, the viability of Ld^AS^ was monitored with the Neubauer chamber and MTT assay. Ld^AS^ was observed to be resistant to the effects of SAG, tolerating drug concentration up to 150 mg/ml, while Ld was susceptible to SAG (IC_50_ 10 μg/ml and IC_90_ 28 μg/ml) (Fig 1B). It was apparent that increasing dose affected the survival of Ld^AS^ to some extent. This result indicated tolerance of high drug concentration was relative rather than absolute, possibly due to high drug pressure that ultimately affect the parasite (Ld^AS^) survival.

### Arsenic exposure increases reduced thiol

**Fig 2:**
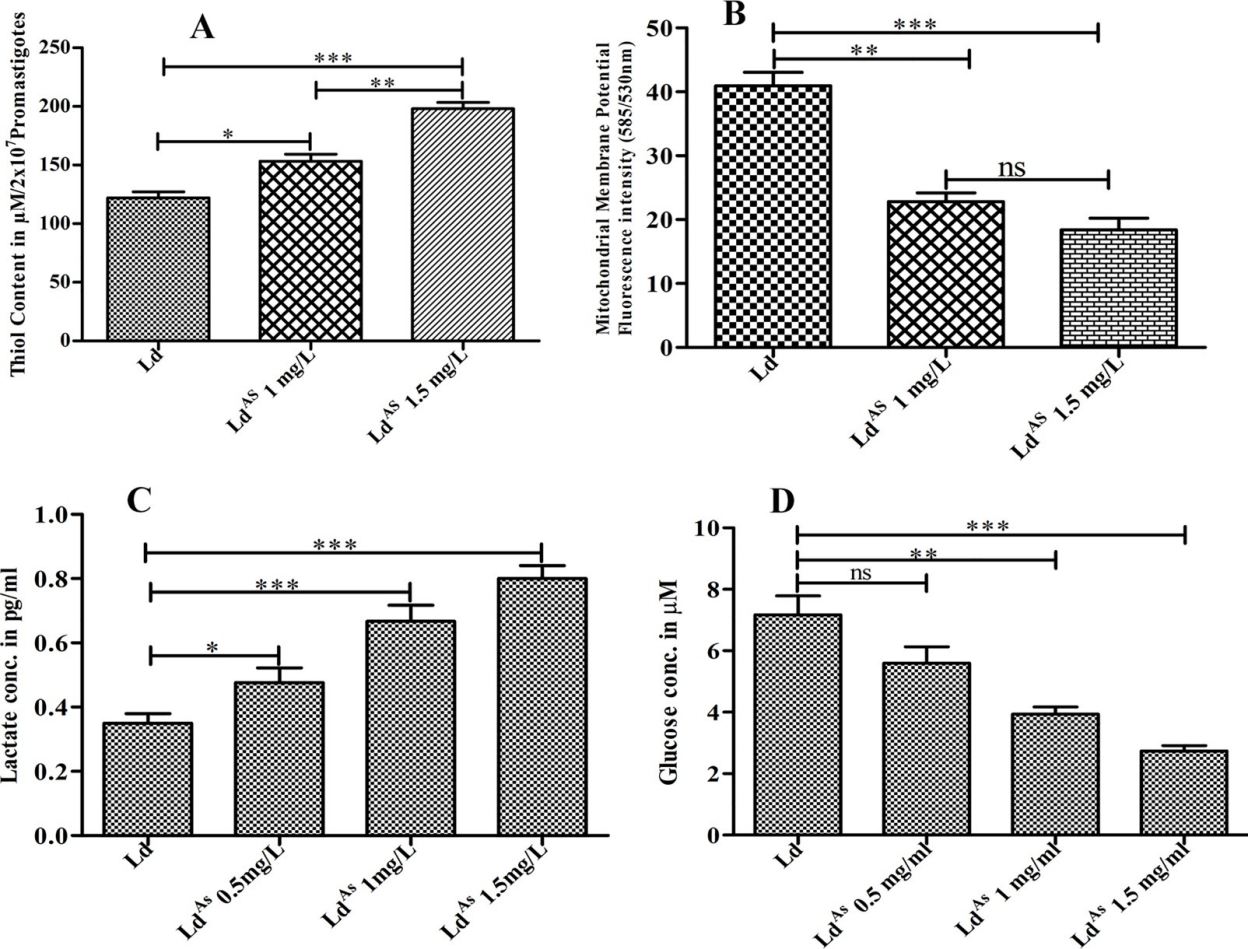
The arsenic exposure to *Leishmania* parasite enhanced the production of reduced thiol and down-regulated the mitochondrial membrane potential (Δψm) as the concentration of reduced thiol was enhanced in Ld^AS^ (1.3-fold in 1 mg/L and 1.7-fold in 1.5 mg/L) compared to Ld, was directly proportional to degree of arsenic exposure (A). The result of Δψm was evaluated using JC-1 dye, that showed 1.8-fold and 2.2-fold depolarization of Δψm in Ld^AS^ (1 and 1.5 mg/L) respectively compared to Ld. The magnitude of depolarization was also directly proportional to quantity of arsenic exposure to *Leishmania* parasite (B). The lactate production and glucose consumption were also increased in Ld^AS^ compared to that of Ld in a dose-dependent fashion. The level of intracellular lactate in parasite (Ld^AS^ & Ld) was estimated by parasites harvesting, homogenizing in assay buffer and analysing by spectrophotometric technique. Ld^AS^ (1 and1.5 mg/L) produced significantly 1.87-fold and 2.28-fold more lactate compare to that of Ld (C). The level of glucose consumption was estimated in Ld^AS^ and Ld, by cultivating parasites in glucose free M199 medium, following centrifugation and evaluating in supernatant by spectrophotometric technique. The glucose levels have been observed 1.79-fold and 2.5-fold less in Ld^AS^ (1 and 1.5 mg/L) respectively than Ld. The results are represented through bars (D) and the data are represented as mean ± SEM (n = 5), the significance of experiments in triplicate, was determined by one-way ANOVA with *Tukey’s post hoc* multiple comparison tests (*P≤0.05; **P≤0.005; ***P≤0.001).

The intracellular reduced thiol of *Leishmania*, a component of trypanothione, has a primary role to nullifying xenobiotic to maintain the redox homeostasis of parasites. The consequences of arsenic on intracellular reduced thiol of Ld^AS^ (1 mg/L and 1.5 mg/L) and Ld were examined using spectrophotometric technique. It was observed 1.7-fold up-regulated (p <0.0005) in Ld^AS^ (1.5 mg/L) compared to that in Ld promastigote (Fig 2A). This increased intracellular reduced thiol content by arsenic (Ld^AS^) acts as a defence mechanism against oxidative stress and helps in parasite growth.

### Arsenic exposure decreases Δψm

For further insight into mitochondria, which has vital role in energy production and survival of the *Leishmania* species, the mitochondrial membrane potential (Δψm) of parasite was evaluated using flow cytometry technique and found to show a significant (p <0.0002) level of depolarization (2.2-fold) of Δψm in Ld^AS^ compared to that of Ld (Fig 2B). This perception revealed that, the Ld^AS^ could subvert their conventional glycolytic pathway to meet the energy demand for their survival.

### Arsenic exposure increases the lactate level and glucose consumption

The lactate level and glucose consumption are directly associated with the growth rate and survival of *Leishmania* parasite via energy currency (ATP) production. The level of intracellular lactate and glucose was measured in Ld^AS^ (1 mg/L and 1.5 mg/L) as well as in Ld by using spectrophotometric technique. The level of lactate produced by Ld^AS^ (1.5 mg/L) was 2.28-fold higher (p <0.0001) compared to that of Ld (Fig 2C), and the glucose level was 2.5-fold lower (p <0.0013) in Ld^AS^ (1.5 mg/L) than that of Ld (Fig 2D), which were predominantly dose-dependent. These results surmise that, Ld^AS^ gradually diverge from their natural glycolytic pathway and possibly use the Warburg effect for their growth and survival.

### Arsenic exposure decreases NO and ROS production

**Fig 3:**
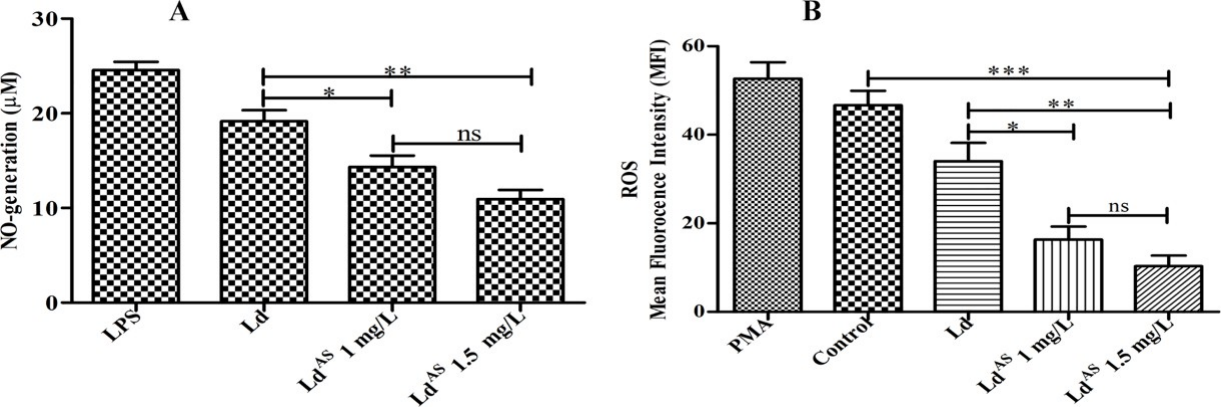
The Ld^AS^ dampened the production of reactive oxygen intermediates compared to that of Ld. The NO production was determined in peritoneal macrophages stimulated with Ld and Ld^AS^ followed by incubation in CO_2_ incubator for 48 hrs, thereafter supernatant was collected, NO was measured using griess reagents (p value <0.0056) (A). The ROS was measured in peritoneal macrophages stimulated with Ld and Ld^AS^ by method as described in “Materials and methods”. The results are shown in a bar diagram (p value <0.0079) (B), where data are expressed as mean ± SEM (n = 5), and significance of experiment in triplicate was determined by one-way ANOVA with *Tukey’s post hoc* multiple comparison tests (*P≤0.05; **P≤0.005; ***P≤0.001).

The reactive intermediate species such as NO and ROS have pivotal role in survival of the intracellular *Leishmania* parasite. The subsequent experiment was performed to check the NO and ROS by spectrophotometric and flow cytometry techniques respectively. The Ld^AS^ stimulated cells produced significantly (p <0.0056) less NO (1.8-fold) (Fig 3A) and significantly (p <0.0079) less ROS (3.2-fold) compared to that of Ld-stimulated cells (Fig 3B). The down-regulated oxidative and nitrosative burst reveals that, arsenic exposure supports the parasite survival, in turn aggressive disease condition.

### Arsenic exposure modulates anti-inflammatory cytokines produced by T lymphocytes

**Fig 4:**
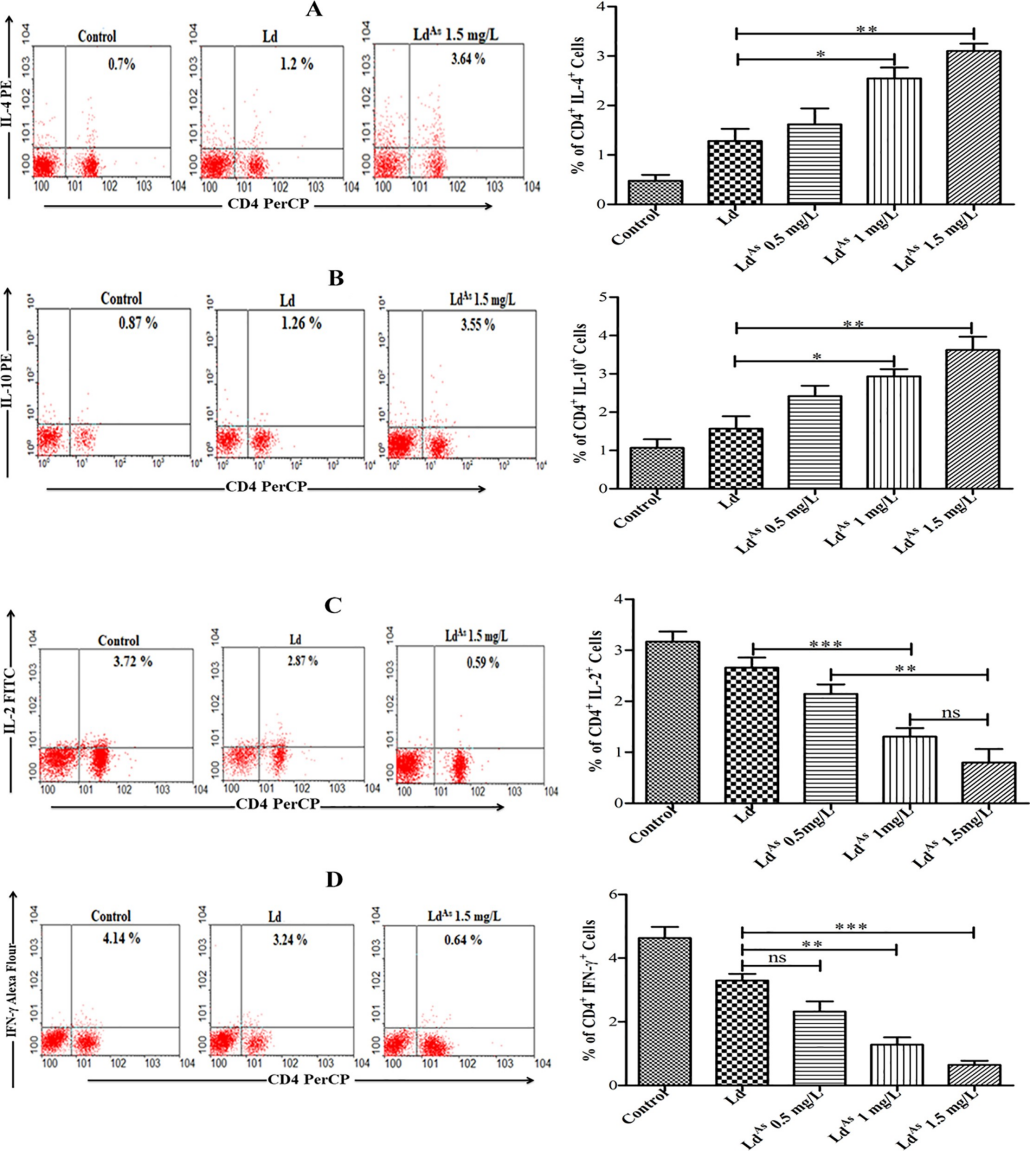
The splenocytes stimulated with Ld^AS^ significantly up-regulated intracellular anti-inflammatory and down-regulated pro-inflammatory cytokines. The anti-inflammatory cytokines IL-4 (p value <0.0033) (A) & IL-10 (p value <0.0034) (B) and pro-inflammatory cytokines IL-2 (p value <0.0049) (C) and IFN-γ (p value <0.0004) (D) were determined by flow cytometry. The data are represented as mean ± SEM (n = 5). All experiments were performed in triplicate and significance was determined by one-way ANOVA with *Tukey’s post hoc* multiple comparison tests (*P≤0.05; **P≤0.005; ***P≤0.001).

**Fig 5:**
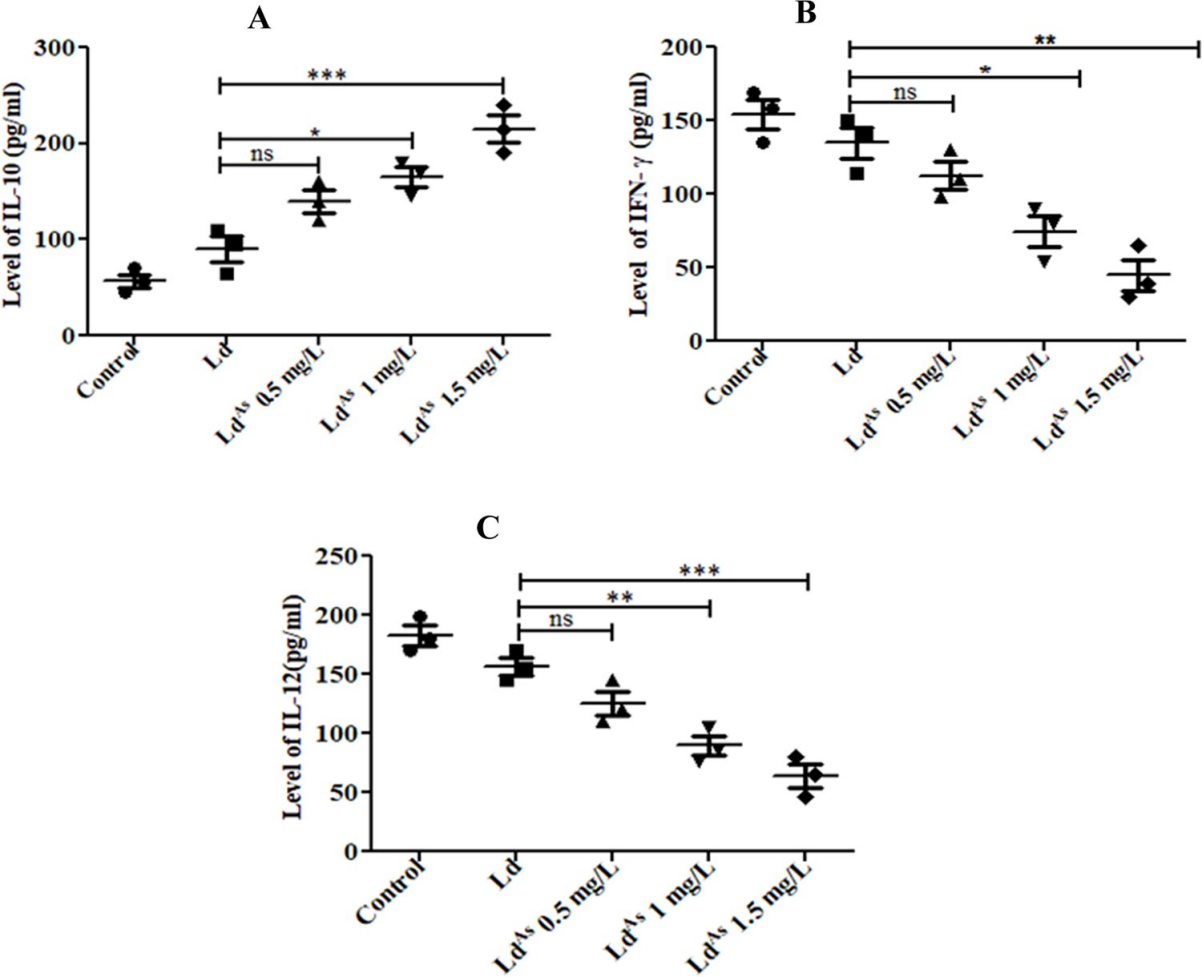
The extra-cellular cytokines, produced by splenocytes stimulated with Ld^AS^ and Ld were also measured by ELISA and results showed, up-regulation of anti-inflammatory cytokine IL-10 (p value <0.0013) (A) and down-regulation of pro-inflammatory cytokines IFN-γ (p value <0.0039) (B) and IL-12 (p value <0.0015) (C) produced by splenocytes stimulated with Ld^AS^ compared to Ld. The data are shown in scatter plot, as mean ± SEM, experiments were performed in triplicate and significance was determined by ANOVA with *Tukey’s post hoc* multiple comparison tests (*P≤0.05; **P≤0.005; ***P≤0.001).

The information on cellular immune response of CD4^+^ T cells (Th1 and Th2) against arsenic exposed *L*. *donovani* in patients is relatively less explored. We, therefore, evaluated both Th1 and Th2 elicited against arsenic exposed *L*. *donovani* parasite in splenocytes of BALB/c mouse. A considerable proportion of CD4^+^ T cells was observed to produce more IL-4 and IL-10, in response to Ld^AS^ group compared to Ld, elicited Th2 cytokines (IL-4, IL-10) advocate for variety of infections and the survival of pathogens inside the host. Altogether, Ld^AS^ group up-regulated IL-4^+^CD4^+^ and IL-10^+^CD^+^ T cells by 2.4 and 2.6-fold (p <0.0033, p<0.0034) (Fig 4A and 4B) respectively compared to arsenic non-exposed group as analysed by flow cytometry. The quantitative analysis of IL-10 by ELISA also showed an increase of 2.26-fold compared to the corresponding value, obtain in splenocytes, stimulated with Ld (Fig 5A).

In contrary, when Th1 cytokines (IL-2 and IFN-γ) in CD4^+^ cells were evaluated, it was observed that the frequencies of IL-2^+^ CD4^+^ and IFN-γ^+^ CD4^+^ cells were decreased 2.8- and 3.2-fold, respectively (p <0.0049, p <0.0004) (Fig 4C and 4D), as analysed by flow cytometry. The IFN-γ level estimated by ELISA also showed 3.37-fold decrease after stimulation with Ld^AS^ compared to that of Ld (Fig 5B).

### Arsenic exposure modulates the cytokines produced by CD14^+^ cells

**Fig 6:**
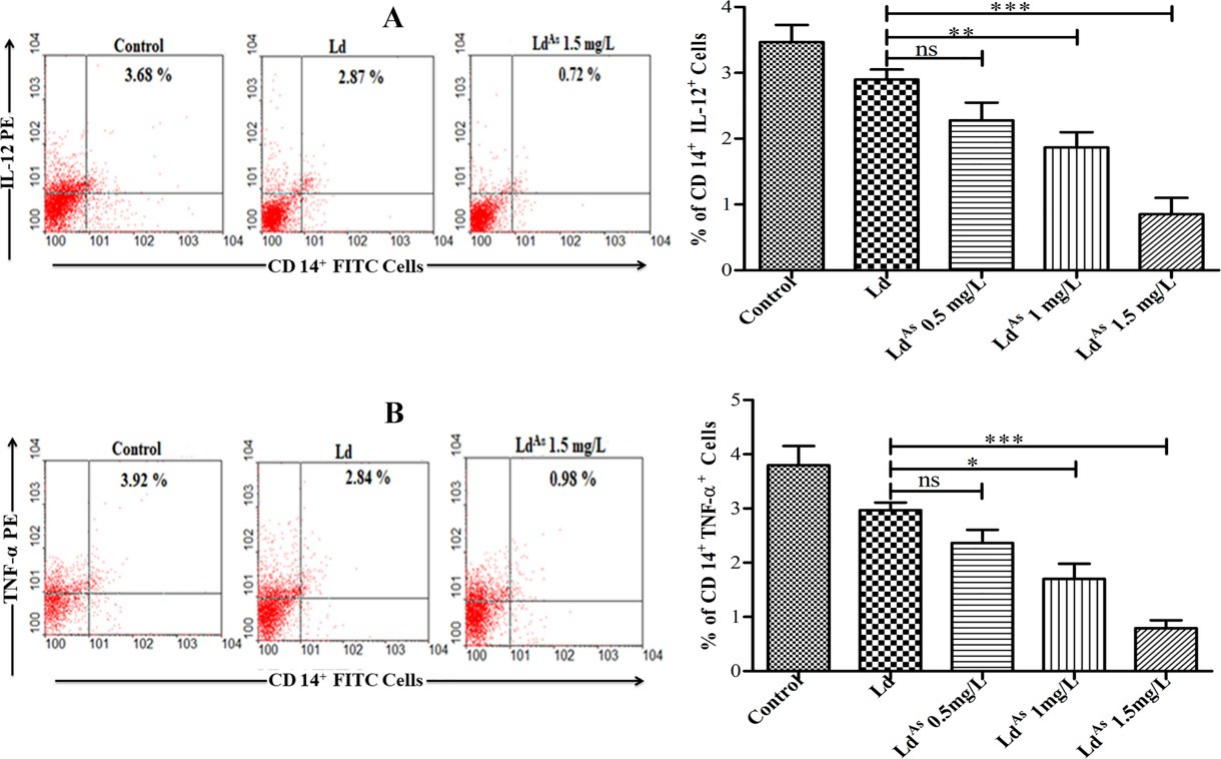
The Ld^AS^ stimulated macrophages (CD14^+^ cells) significantly down-regulated the intracellular pro-inflammatory cytokines. For this, the splenocytes derived macrophages, stimulated with Ld^AS^ & Ld, stained with surface FITC labelled CD14^+^ and intracellular cytokines stained with individually PE labelled IL-12 & TNF-α antibodies were analysed by flow cytometry as described in “Material and methods”. The data of experiment in triplicate represent mean ± SEM (n = 5) and significance was determined by one-way ANOVA with *Tukey’s post hoc* multiple comparison tests (*P≤0.05; **P≤0.005; ***P≤0.001).

The pro-inflammatory cytokines such as IL-12 and TNF-α, produced by macrophages (CD14^+^ cells) [25], upon activation, supports the immune response to eliminate the intracellular *L*. *donovani* infection. The observed levels of IL-12 and TNF-α in CD14^+^ cells in response to Ld^AS^ were found to be significantly down-regulated by 2.2-fold (p<0.0013) and 2.34-fold (p <0.0005) respectively compared to that of Ld (Fig 6A and 6B), as it was analysed by flow cytometry. The IL-12 level, estimated by ELISA also showed 2.21-fold decrease after stimulation with Ld^AS^ compared to that of Ld (Fig 5C).

Such findings in CD4^+^ and CD14^+^ cells suggest that immune response triggered by arsenic exposed *L*. *donovani* directs predominantly Th2 type of immune response, which can exacerbate the disease.

## Discussion

In view of published report of arsenic contamination in the ground drinking water up to 1861 ppb in endemic area of visceral leishmaniasis, Bihar, India [36], residents may consume high concentration of arsenic. Although a majority of the arsenic may be cleared from the blood within hours [37], due to the regular intake of high concentrations of the element, arsenic deposition can be expected in the liver, spleen and bone marrow, leading to increased levels compared to that in blood [6], where the virulent *Leishmania donovani* form resides during infection. Therefore, during this *in vitro* study, the authors have standardized the laboratory culture of *Leishmania donovani* parasites with a lower concentration (0.5 to 1.5 mg/L) of arsenic, which was found to be optimal for immunomodulation and infection establishment.

The arsenic exposure to *Leishmania donovani* forced the parasites to develop resistance against antimony, and the exposed *Leishmania* strain tolerated a drug (SAG) at concentrations between 20 to 150 μg/ml (Fig 1B). This finding is supported by earlier work on arsenic-exposed *Leishmania* parasites [6,38]. Available reports also suggest that the antimony must be reduced from a pentavalent configuration to a trivalent, the one present after the intake of arsenic in the cells [39,40]. Since both arsenic and antimony are from the same group in the periodic table and are transported in the cells by the same mechanism, it was hypothesized that they both might have a chance for cross-resistance [41]. Additionally, the enhanced reduced thiol may also efflux the drug [11,14,27]. Although not conclusively worked out, these reasons may be amongst multiple causes for arsenic-exposed *Leishmania* resistance to antimony.

To circumvent the effects of arsenic in the promotion of *Leishmania* infection (the antigenic capacity of arsenic-exposed resistance we examined here), we found ample evidence that arsenic exposure to a parasite can result in increased infectivity (Fig 1A) in the BALB/c mouse-derived macrophages compared to that in cells with no exposure. Reports that the parasite increases the likelihood of its survival in the host by affecting the trypanothione synthase (TSH2) pathway (resulting in the production of major reduced thiol and affecting the abrupt release of ROS) are available [27]. The up-regulated level of the reduced thiol and trypanothione pathway were also reported for SAG, amphotericin-B (AMB) and miltefosine resistance [42,43,44]. Consistent with these findings, we also observed significantly up-regulated levels of reduced thiol production (1.7-fold more) in the arsenic-exposed *Leishmania* parasite compared to that in the unexposed parasite (Fig 2A). The suppressed reactive nitrogen and oxygen intermediates (NO, ROS) (Fig 3A and 3B) observed in this study logically support a higher infectivity by the arsenic exposed parasite. However, the loss of the mitochondrial membrane potency leads to the use of ATP for energy production, as we found that a considerable level of depolarization of the membrane potential had occurred in Ld^AS^ compared to that in unexposed Ld (Fig 2B). This finding is also supported by some reports suggesting the role of arsenic in the depolarization of the mitochondrial membrane potential [16,45]. As the entry of arsenite in the glycolytic pathway was reported to affect the cellular energy metabolism [46,47], this observation raises the question regarding the increased infection due to the arsenic effect. One argument in support of the hypothesis might be explained by the accumulation of lactate (Fig 2C), which can indicate a conversion from pyruvate rather than acetyl-CoA that could not enter adequately into the TCA cycle of mitochondria due to the depolarization of the mitochondria membrane potential. The overproduction of lactate could be converted back to glucose by gluconeogenesis, as we found high levels of glucose consumption in arsenic-exposed *Leishmania* parasites (Fig 2D). Thus, the rate of glycolysis might be many fold higher and rapidly followed by the Warburg effect to increase infectivity [48,49,50].

Due to all these changes in arsenic-exposed parasites, it was interesting to examine the effect of the parasites on immunomodulation in the host, especially when *Leishmania* was not exposed to arsenic. The macrophage activation and elimination of the intracellular *Leishmania* parasites is highly dependent on the generation of reactive oxygen and nitrogen species. Both IL-12 and TNF-α produced predominantly by APCs are reported to have a major impact in the generation of ROS and NO. We observed that the expression levels of both ROS and NO were significantly (p<0.0079, p<0.0056, respectively) down-regulated in macrophage cells stimulated by Ld^AS^ compared to that in cells stimulated by Ld and the control group. We also noticed that the frequency of IL-12- and TNF-α-producing CD14^+^ cells in Ld^AS^-stimulated splenocytes was down-regulated (Fig 6A and 6B) compared to the levels in Ld-stimulated cells and the control group, which may have led to an immunosuppressive state in the host. We were able to observe that splenocytes stimulated with arsenic-exposed parasites had a reduced frequency of cells producing IL-2 and IFN-γ (p<0.0049, p<0.0004, respectively) (Figs 4C, 4D and 5B). In contrast, the levels of Th2 cytokines such as IL-4 and IL-10 became high after arsenic exposure to Ld (Figs 4A, 4B and 5A) compared to those in unexposed Ld stimulated cells. Thus, arsenic-exposed *Leishmania* parasites suppressed the early production of IL-2, IL-12 and TNF-α with the under-expression of ROS and NO by CD14^+^ cells. The expression of IFN-γ was significantly down-regulated (p<0.0004) and the expression of IL10 and IL-4 was significantly up-regulated (p<0.0034; p<0.0033, respectively) based on the percentage of CD4^+^ cells analysed by flow cytometry. These results are in agreement with the quantitative analysis by ELISA in cells stimulated with Ld^AS^ as reported in other studies [32,51,52]. These *in vitro* findings suggest the possibility of immunomodulation and infection due to arsenic-exposed parasites, which warrants further investigation using arsenic-exposed intracellular amastigotes since the likelihood of drinking arsenic-contaminated water is very high in the visceral leishmaniasis endemic area in the Indian sub-continent, where the disease has rapidly worsened. Thus, it was revealed that arsenic intake leads to changes in the biology of *Leishmania* parasites, likely conferring a severe immunosuppressive state and infection in VL. This study will be valuable for the control of visceral leishmaniasis resistance by launching an awareness programme to provide arsenic-free water.

## Supporting information

S1 Fig The viability of *Leishmania* parasite was assessed against SAG. For this, peritoneal macrophages of BALB/c mice were isolated, cultured and incubated in CO_2_ incubator for 48 hrs. Thereupon, the cells were infected with Ld and further incubated in CO_2_ incubator. Subsequently, different concentrations of drug (SAG) were added to respective wells and untreated well was kept as a control. The observed result is shown in S1 Fig.
(TIF)

S2 Fig The splenocytes, stimulated with LdAS showed significantly up-regulated intracellular anti-inflammatory cytokine like IL-4. For this, splenocytes derived T cells were stained with surface PerCP labelled CD4+ and intracellular PE labelled IL-4 antibodies followed by flow cytometry analysis and observed result is shown in S2 Fig.
(TIF)

S3 Fig The LdAS stimulated macrophages (CD14+ cells) showed down-regulated intracellular pro-inflammatory cytokines. For this, macrophage cells were stimulated with Ld^AS^ & Ld, stained with surface FITC labelled CD14^+^ and intracellular PE labelled TNF-α antibodies and analysed by flow cytometry as described in “Material and methods”. Result is shown in S3 Fig.
(TIF)

S4 Fig The NO production was determined in peritoneal macrophages, stimulated with Ld and LdAS and followed by incubation in CO_2_ incubator. Thereafter, supernatant was collected and NO was measured using griess reagents. Result is shown in S4 Fig.
(TIF)

S5 Fig The concentration of reduced thiol was enhanced in LdAS compared to Ld, as measured by Elman’s regent. The observed data is shown in S5 Fig.
(TIF)
