# Supplementary figures and images for: Modulation of the immune response and infection pattern to Leishmania donovani in visceral leishmaniasis due to arsenic exposure: An in vitro study
Source: PLoS One. 2019 Feb 5;14(2):e0210737. doi: 10.1371/journal.pone.0210737 (PMC6363178; doi:10.1371/journal.pone.0210737)

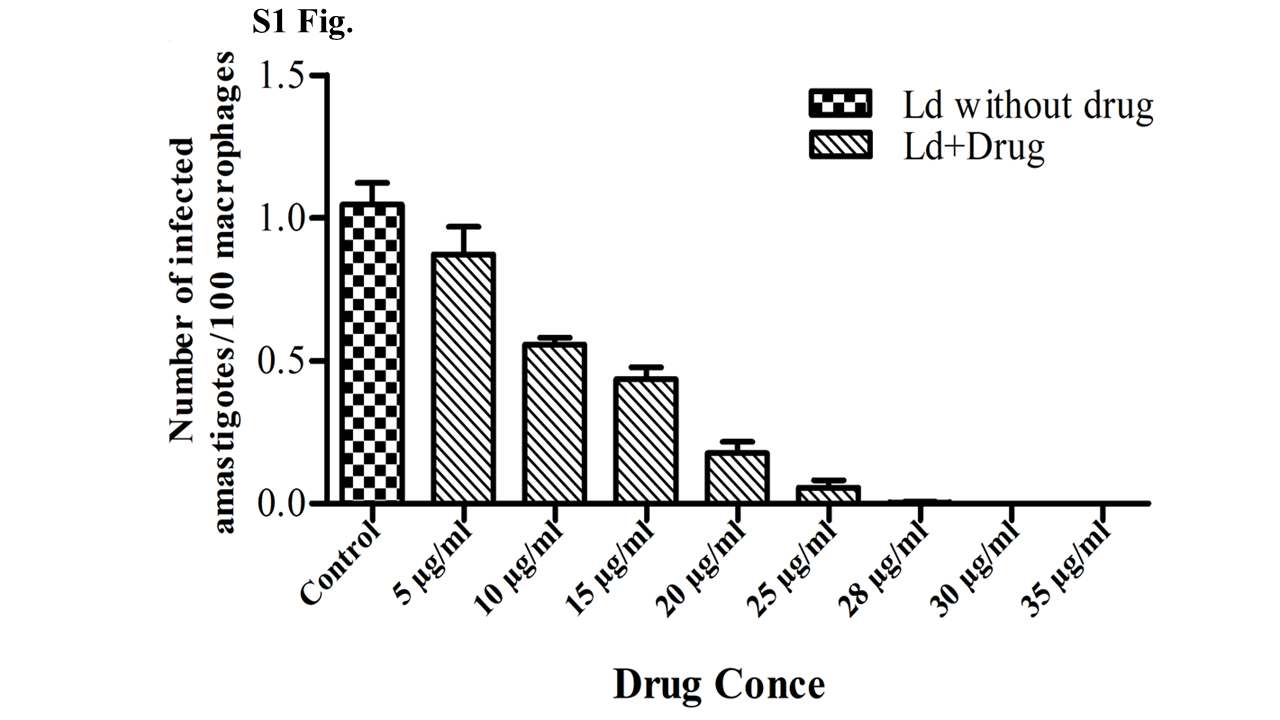

Supplement: S1 Fig — For this, peritoneal macrophages of BALB/c mice were isolated, cultured and incubated in CO2 incubator for 48 hrs. Thereupon, the cells were infected with Ld and further incubated in CO2 incubator. Subsequently, different concentrations of drug (SAG) were added to respective wells and untreated well was kept as a control. The observed result is shown in S1 Fig. (TIF) [file pone.0210737.s001.tif]

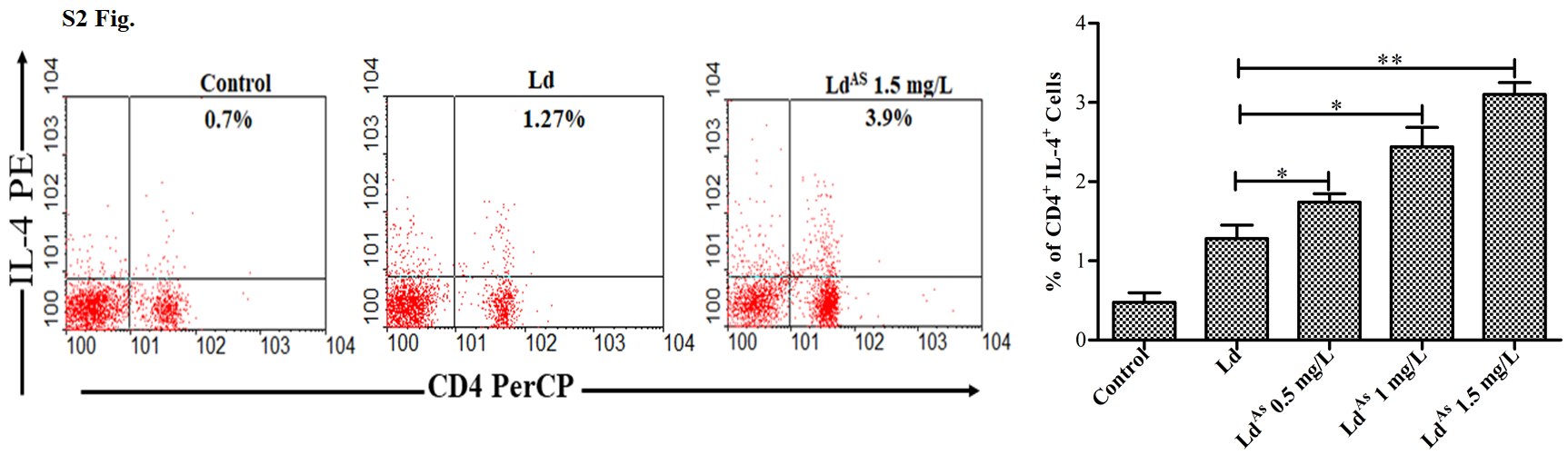

Supplement: S2 Fig — For this, splenocytes derived T cells were stained with surface PerCP labelled CD4+ and intracellular PE labelled IL-4 antibodies followed by flow cytometry analysis and observed result is shown in S2 Fig. (TIF) [file pone.0210737.s002.tif]

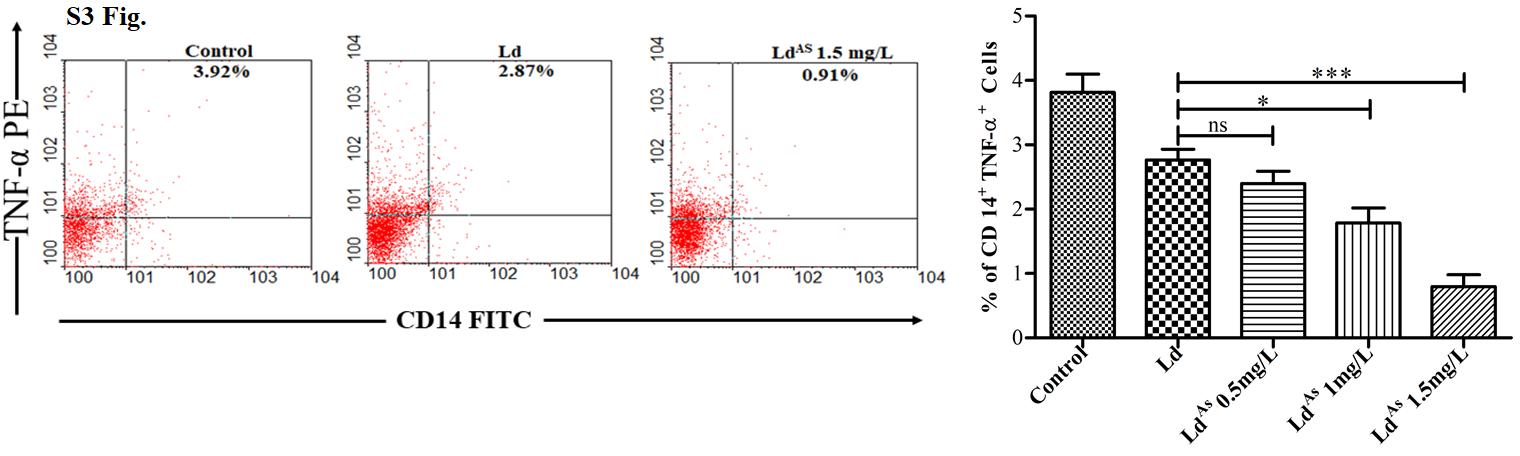

Supplement: S3 Fig — For this, macrophage cells were stimulated with LdAS & Ld, stained with surface FITC labelled CD14+ and intracellular PE labelled TNF-α antibodies and analysed by flow cytometry as described in “Material and methods”. Result is shown in S3 Fig. (TIF) [file pone.0210737.s003.tif]

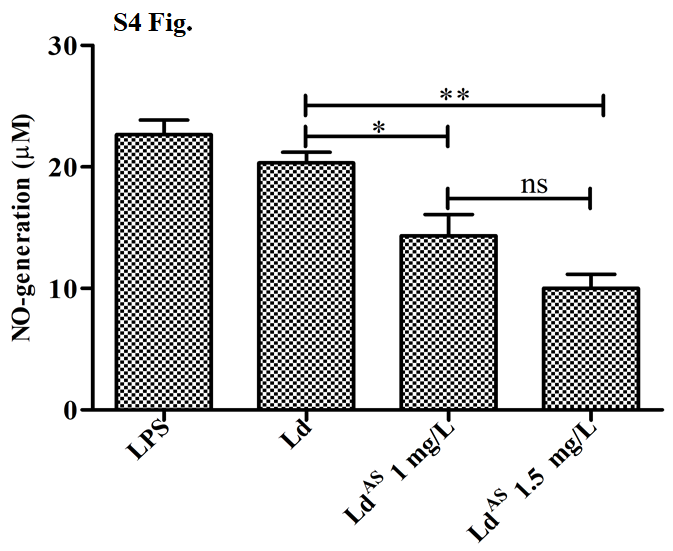

Supplement: S4 Fig — Thereafter, supernatant was collected and NO was measured using griess reagents. Result is shown in S4 Fig. (TIF) [file pone.0210737.s004.tif]

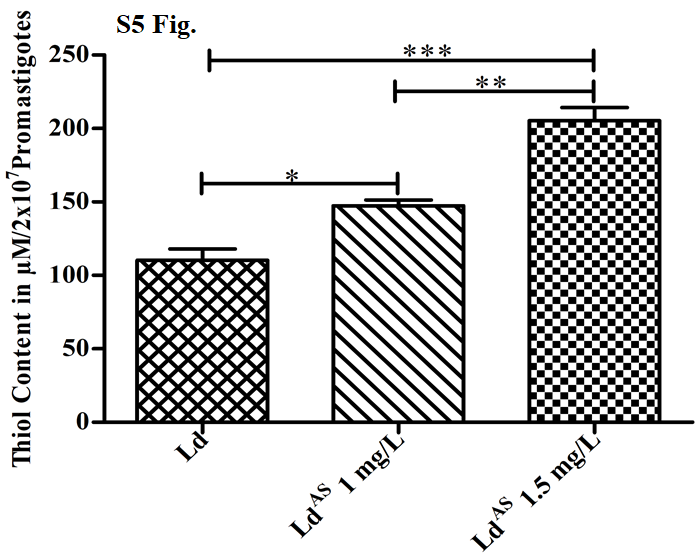

Supplement: S5 Fig — The observed data is shown in S5 Fig. (TIF) [file pone.0210737.s005.tif]
